# Supplementary material for: Assessing Upper Limb Function in Breast Cancer Survivors Using Wearable Sensors and Machine Learning in a Free-Living Environment
Source: Sensors (Basel). 2023 Jul 2;23(13):6100. doi: 10.3390/s23136100 (PMC10347074; doi:10.3390/s23136100)

Supplementary material 1: Figure representing raw acceleration signal (m/s²) and the manual video annotation with the functional, non-functional and unknown label.

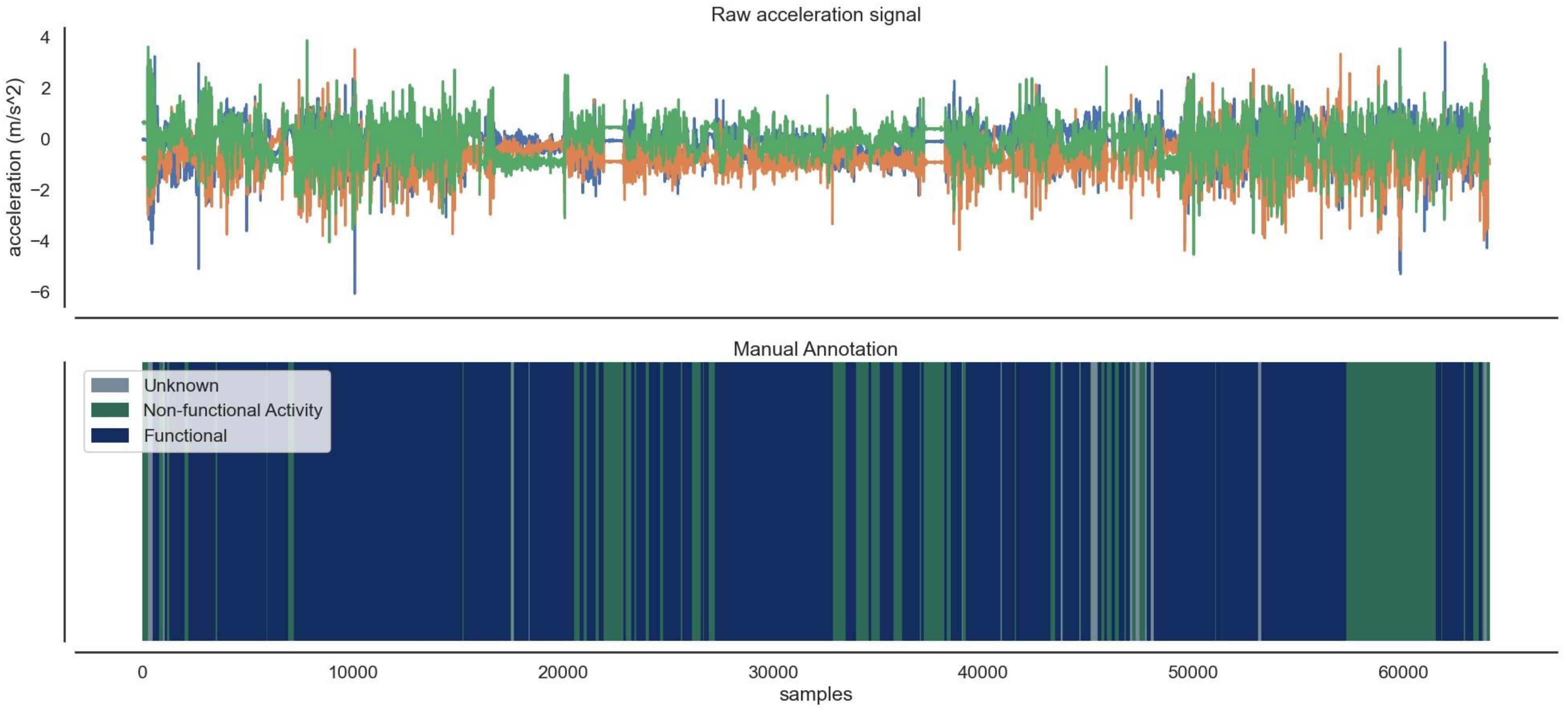

Supplement: Supplementary file 1 [file sensors-23-06100-s001.zip › sensors-2459270-supplementary.pdf]
